# Use of new approach methodologies (NAMs) to meet regulatory requirements for the assessment of industrial chemicals and pesticides for effects on human health

**DOI:** 10.3389/ftox.2022.964553

**Published:** 2022-09-01

**Authors:** Andreas O. Stucki, Tara S. Barton-Maclaren, Yadvinder Bhuller, Joseph E. Henriquez, Tala R. Henry, Carole Hirn, Jacqueline Miller-Holt, Edith G. Nagy, Monique M. Perron, Deborah E. Ratzlaff, Todd J. Stedeford, Amy J. Clippinger

**Affiliations:** ^1^ PETA Science Consortium International e.V., Stuttgart, Germany; ^2^ Safe Environments Directorate, Healthy Environments and Consumer Safety Branch, Health Canada, Ottawa, ON, Canada; ^3^ Pest Management Regulatory Agency, Health Canada, Ottawa, ON, Canada; ^4^ Corteva Agriscience, Indianapolis, IN, United States; ^5^ Office of Pollution Prevention and Toxics, US Environmental Protection Agency, Washington, DC, United States; ^6^ Scientific and Regulatory Affairs, JT International SA, Geneva, Switzerland; ^7^ Bergeson & Campbell PC, Washington, DC, United States; ^8^ Office of Pesticide Programs, US Environmental Protection Agency, Washington, DC, United States

**Keywords:** new approach methodologies (NAMs), *in vitro*, *in silico*, risk assesment, toxicity testing, industrial chemicals, pesticides

## Abstract

New approach methodologies (NAMs) are increasingly being used for regulatory decision making by agencies worldwide because of their potential to reliably and efficiently produce information that is fit for purpose while reducing animal use. This article summarizes the ability to use NAMs for the assessment of human health effects of industrial chemicals and pesticides within the United States, Canada, and European Union regulatory frameworks. While all regulations include some flexibility to allow for the use of NAMs, the implementation of this flexibility varies across product type and regulatory scheme. This article provides an overview of various agencies’ guidelines and strategic plans on the use of NAMs, and specific examples of the successful application of NAMs to meet regulatory requirements. It also summarizes intra- and inter-agency collaborations that strengthen scientific, regulatory, and public confidence in NAMs, thereby fostering their global use as reliable and relevant tools for toxicological evaluations. Ultimately, understanding the current regulatory landscape helps inform the scientific community on the steps needed to further advance timely uptake of approaches that best protect human health and the environment.

## 1 Introduction

Regulatory agencies are tasked with ensuring protection of human health and the environment, and implementing various processes for achieving this goal. Legal frameworks that do not require upfront toxicological testing have relied heavily on chemical evaluations using analogue read across and grouping based on chemical categories, while others with upfront testing requirements have relied on prescribed checklists of toxicity tests, often using animals to fulfill the required testing. However, scientific advancements have led to investments in the development, implementation, and acceptance of reliable and relevant new approach methodologies (NAMs). NAMs are defined as any technology, methodology, approach, or combination that can provide information on chemical hazard and risk assessment without the use of animals, including *in silico*, *in chemico*, *in vitro*, and *ex vivo* approaches (ECHA, 2016b; EPA, 2018d). NAMs are not necessarily newly developed methods, rather, it is their application to regulatory decision making or replacement of a conventional testing requirement that is new.

Regulatory agencies worldwide have recognized the importance of the timely uptake of fit for purpose NAMs for hazard and risk assessment and are introducing flexible, efficient, and scientifically sound processes to establish confidence in the use of NAMs for regulatory decision-making (van der Zalm et al., 2022; Ingenbleek et al., 2020). The use of NAMs has been prioritized because of their ability to efficiently generate information that, once established to be as or more reliable and relevant than the conventional testing requirement, may be used to make regulatory decisions that protect human health. NAMs can mimic human biology and provide mechanistic information about how a chemical may cause toxicity in humans. They can also be used to inform population variability, for example, by rapidly identifying susceptible subpopulations from potential exposures in fence line communities or workers, and by allowing for the consideration of individualized health risks and the generation of data tailored to people with pre-existing conditions or those more sensitive to certain chemicals (EPA, 2020e).

This article describes opportunities for and examples of the use of NAMs in regulatory submissions for industrial chemicals and pesticides in the United States (US), Canada, and the European Union (EU). For industrial chemicals, it includes the US Environmental Protection Agency (EPA)’s Office of Pollution Prevention and Toxics (OPPT), the US Consumer Products Safety Commission (CPSC), Health Canada (HC)’s Healthy Environments and Consumer Safety Branch (HECSB), and the European Chemicals Agency (ECHA). For pesticides and plant protection products (PPP), it highlights the EPA’s Office of Pesticide Programs (OPP), HC’s Pest Management Regulatory Agency (PMRA), and the European Food Safety Authority (EFSA). This article also provides examples of collaborations, across sectors and borders, to build scientific, regulatory, and public confidence in the use of NAMs for the protection of human health, and to reach the ultimate goal of global acceptance. Tables 1, 2 summarize some of the guidance, strategic plans, and other helpful documentation related to the implementation of NAMs. While this article addresses the assessment of human health effects of industrial chemicals and pesticides in the US, Canada, and the EU, similar collaborative efforts and opportunities to use NAMs in regulatory submissions exist in other sectors and countries. Furthermore, many of the discussed actions and efforts also likely apply to other types of chemicals and to ecotoxicological effects.

**TABLE 1:** US, Canada, and EU: industrial chemicals and household products.

Agency	Strategic plans, guidance, and other documentation for the implementation of NAMs referenced in this manuscript
EPA OPPT	• Interim science policy: use of alternative approaches for skin sensitization as a replacement for laboratory animal testing EPA, (2018b)
	• Strategic plan to promote the development and implementation of alternative test methods within the TSCA program EPA, (2018d)
	• Utility of *In Vitro* Bioactivity as a Lower Bound Estimate of *In Vivo* Adverse Effect Levels and in Risk-Based Prioritization Paul Friedman et al. (2020) ^a^
	• List of alternative test methods and strategies (or new approach methodologies [NAMs]), Second update: 4 February 2021 EPA, (2021d)
	• New approach methods work plan, reducing use of animals in chemical testing EPA, (2021e) ^b^
	• A WoE Approach for Evaluating, in Lieu of Animal Studies, the Potential of a Novel Polysaccharide Polymer to Produce Lung Overload Ladics et al. (2021)
CPSC	• Recommended Procedures Regarding the CPSC’s Policy on Animal Testing (16 CFR Part 1500)
	• Guidance on Alternative Test Methods and Integrated Testing Approaches CPSC, (2022)
HC HECSB	• Fact sheet series: Topics in risk assessment of substances under CEPA HC, (2016b)
	• Guidance document for the notification and testing of new chemicals and polymers HC, (2021c)
	• Canadian regulatory perspective on next generation risk assessments for pest control products and industrial chemicals Bhuller et al. (2021)
	• Utility of *In Vitro* Bioactivity as a Lower Bound Estimate of *In Vivo* Adverse Effect Levels and in Risk-Based Prioritization Paul Friedman et al. (2020)
	• Science approach documents HC, (2016c), HC, (2021f), HC, (2022c), HC, (2021f), HC, (2022c)
ECHA	• How to use alternatives to animal testing to fulfil the information requirements for REACH registration ECHA, (2016a)
	• Read-across assessment framework ECHA, (2017)
	• 4th report on the use of alternatives to testing on animals for REACH ECHA, (2020)
	• Utility of *In Vitro* Bioactivity as a Lower Bound Estimate of *In Vivo* Adverse Effect Levels and in Risk-Based Prioritization Paul Friedman et al. (2020)
	• Skin sensitization ECHA, (2021)

a EPA Office of Research and Development (ORD) involved.

b Applicable to all EPA offices.

CEPA, Canadian Environmental Protection Act; CFR, Code of Federal Regulations; CPSC, Consumer Products Safety Commission; ECHA, European Chemicals Agency; EPA OPPT, Environmental Protection Agency Office of Pollution Prevention and Toxics; HC HECSB, Health Canada Healthy Environments and Consumer Safety Branch; NAM, new approach methodologies; TSCA, Toxic Substances Control Act; WoE, weight-of-evidence.

**TABLE 2:** US, Canada, and EU: pesticides and plant protection products.

Agency	Strategic plans, guidance, and other documentation for the implementation of NAMs referenced in this manuscript
EPA OPP	• Guidance for waiving or bridging of acute EPA, (2012); EPA, (2016a); EPA, (2020c) and repeat dose EPA, (2013b) toxicity tests for pesticides (for formulations and single-active ingredients)
	• Use of an alternate testing framework for classification of eye irritation potential of EPA pesticide products EPA, (2015)
	• Process for evaluating & implementing alternative approaches to traditional *in vivo* acute toxicity studies for FIFRA regulatory use EPA, (2016b)
	• Interim science policy: Use of alternative approaches for skin sensitization as a replacement for laboratory animal testing EPA, (2018b)
	• Recommendation on test readiness criteria for new approach methods in toxicology: Exemplified for DNT Bal-Price et al, (2018a) ^a^
	• Utility of *In Vitro* Bioactivity as a Lower Bound Estimate of *In Vivo* Adverse Effect Levels and in Risk-Based Prioritization Paul Friedman et al. (2020) ^a^
	• New approach methods work plan, reducing use of animals in chemical testing EPA, (2021e) ^b^
	• Retrospective analysis of the dermal absorption “triple pack” data Allen et al. (2021)
	• Performance of the GHS Mixtures Equation for Predicting Acute Oral Toxicity Hamm et al. (2021)
	• Integration of toxicodynamic and toxicokinetic new approach methods into a WoE analysis for pesticide DNT assessment Dobreniecki et al. (2022)
	• ReCAAP: A reporting framework to support a weight of evidence safety assessment without long-term rodent bioassays EPA, (2020f); Hilton et al. (2022)
HC PMRA	• Guidance for waiving or bridging of mammalian acute toxicity tests for pesticides HC, (2013a); Acute Dermal Toxicity Study Waiver HC, (2017)
	• PMRA’s 2016–2021 strategic plan HC, (2016d)
	• Canadian regulatory perspective on next generation risk assessments for pest control products and industrial chemicals Bhuller et al. (2021)
	• Guidance for developing datasets for conventional pest control product applications HC, (2021d)
	• ReCAAP: A reporting framework to support a weight of evidence safety assessment without long-term rodent bioassays Hilton et al. (2022)
EFSA	• Guidance on dermal absorption EFSA et al. (2017)
	• OECD/EFSA workshop on DNT: The use of non-animal test methods for regulatory purposes Fritsche et al. (2017)
	• Reconnecting exposure, toxicokinetics and toxicity in food safety: OpenFoodTox and TKplate for human health, animal health and ecological risk assessment Dorne et al. (2018)
	• Recommendation on test readiness criteria for new approach methods in toxicology: Exemplified for DNT Bal-Price et al. (2018a)
	• Workshop on *in vitro* comparative metabolism studies in regulatory pesticide risk assessment EFSA, (2019)
	• Advancing human health risk assessment Lanzoni et al. (2019)
	• Utility of *In Vitro* Bioactivity as a Lower Bound Estimate of *In Vivo* Adverse Effect Levels and in Risk-Based Prioritization Paul Friedman et al. (2020)
	• Development of IATA case studies on DNT risk assessment EFSA PPR Panel et al. (2021)
	• EFSA Strategy 2027 EFSA, (2021)
	• Development of a Roadmap for Action on New Approach Methodologies in Risk Assessment Escher et al. (2022)

a EPA Office of Research and Development (ORD) involved.

b Applicable to all EPA offices.

EFSA, European Food Safety Authority; EPA OPP, Environmental Protection Agency Office of Pesticide Programs; FIFRA, Federal Insecticide, Fungicide, and Rodenticide Act; HC PMRA, Health Canada Pest Management Regulatory Agency; NAM, new approach methodologies. ReCAAP, Rethinking Chronic toxicity and Carcinogenicity Assessment for Agrochemicals Project.

## 2 Overarching activities to advance the implementation of NAMs

### 2.1 International collaboration

The Organisation for Economic Co-operation and Development (OECD) publishes guidelines for the assessment of chemical effects on human health and the environment. Under the mutual acceptance of data (MAD) agreement among the 38 OECD member countries, which aims to reduce duplicate testing, “…data generated in the testing of chemicals in an OECD member country in accordance with OECD Test Guidelines (TG) and OECD Principles of Good Laboratory Practice (GLP), shall be accepted in other member countries” (OECD, 2019). A portion of the nearly 100 OECD test guidelines describe *in chemico, in vitro,* or *ex vivo* methods that are accepted by certain regulatory agencies for the testing of various types of chemicals. At their discretion, agencies can decide which OECD test guidelines to require and whether to accept non-OECD guideline methods (OECD, 2019). Building toward regulatory implementation of non-guideline methods, parallel OECD efforts are working to advance the development of best practices, guidance, data integration and evaluation frameworks such as Integrated Approaches to Testing and Assessment (IATA) and Adverse Outcome Pathways (AOPs).

The International Cooperation on Alternative Test Methods (ICATM) was originally established in 2009 by the US Interagency Coordinating Committee for the Validation of Alternative Methods (ICCVAM), HC, the EU Reference Laboratory for alternatives to animal testing (EURL ECVAM), and the Japanese Center for the Validation of Alternative Methods (JaCVAM) to facilitate cooperation among national validation organizations. Since its establishment, Korea (KoCVAM) has signed the agreement and China, Brazil (BraCVAM), and Taiwan participate in ICATM activities. In 2019, Canada established the Canadian Centre for the Validation of Alternative Methods (CaCVAM). Each group works in-country and collaboratively to advance NAMs. For example, the Tracking System for Alternative Methods (TSAR), an overview of non-animal methods that have been proposed for regulatory safety or efficacy testing of chemicals or biological agents, was established and provided by EURL ECVAM (EURL ECVAM, n.d.).

In 2016, ECHA organized a workshop on NAMs in Regulatory Science, which was attended by 300 stakeholders to discuss the use of NAMs for regulatory decision making (ECHA, 2016b). Since 2016, EPA, HC, and ECHA have held workshops to discuss the development and application of NAMs for chemical assessment as part of an international government-to-government initiative titled “Accelerating the Pace of Chemical Risk Assessment” (APCRA) (EPA, 2021a). EPA and HC further collaborated through the North American Free Trade Agreement (NAFTA; in 2020, NAFTA was replaced by the US-Mexico-Canada Agreement (USMCA)) Technical Working Group (TWG) on Pesticides and through the Canada-US Regulatory Co-operation Council (RCC). The RCC was a regulatory partnership between the pesticides regulating department and offices of HC and EPA that has facilitated the alignment of both countries’ regulatory approaches, while advancing efforts to reduce and replace animal tests (ITA, n.d.; HC, 2020). The NAFTA TWG on Pesticides and the RCC included specific work plans and priority areas along with accountability for deliverables (NAFTA TWG, 2016).

The development and implementation of NAMs within regulatory agencies relies heavily on collaboration with a variety of stakeholders, including other offices and departments within the same agency, other national and international agencies, as well as industry representatives, method developers, academics, and non-profit/non-governmental organizations. For example, within EPA, there is substantial cross-talk between OPP and OPPT (both of which are a part of the Office of Chemical Safety and Pollution Prevention (OCSPP)) as well as the Office of Research and Development (ORD). Agencies also consult with external peer-review panels, such as science advisory boards or committees, which provide independent scientific expertise on various topics. The exchange with external stakeholders provides diverse perspectives and experiences with different NAMs. Several of these collaborations have led to journal publications, presentations at national and international meetings, and webinars. For example, since 2018, EPA has partnered with PETA Science Consortium International e.V. and the Physicians Committee for Responsible Medicine to host a webinar series on the “Use of New Approach Methodologies (NAMs) in Risk Assessment” which brings together expert speakers and attendees from around the world to discuss the implementation of NAMs (PSCI, n.d.). EPA’s OCSPP and ORD also held conferences on the state of the science for using NAMs in 2019 and 2020 and are currently planning the next conference for October 2022 (EPA, 2019a; EPA, 2020b).

### 2.2 National roadmaps or work plans to guide and facilitate the implementation of NAMs

#### 2.2.1 United States

Several US agencies have roadmaps or work plans to guide and facilitate the implementation of NAMs for testing industrial chemicals or pesticides. For example*,* following publication of the EPA-commissioned National Resource Council (NRC) report titled “Toxicity Testing in the 21st Century: A Vision and A Strategy” (NRC, 2007), EPA released a strategic plan that provided a framework for implementing the NRC’s vision, which incorporates new approaches into toxicity testing and risk assessment practices with less reliance on conventional apical approaches (EPA, 2009). Furthermore, in June 2020, EPA’s OCSPP and ORD published a NAM Work Plan (updated in December 2021) that describes primary objectives and strategies for reducing animal testing through the use of NAMs while ensuring protection of human health and the environment (EPA, 2021e). It highlights the importance of communicating, collaborating, providing training on NAMs, establishing confidence in NAMs, and developing metrics for assessing progress.

In 2018, the 16 US federal agencies that comprised ICCVAM (including EPA and CPSC) published a strategic roadmap to serve as a guide for agencies and stakeholders seeking to adopt NAMs for chemical safety and risk assessments (ICCVAM, 2018). The ICCVAM strategic roadmap emphasizes three main components: 1) connecting agency and industry end users with NAM developers to ensure the needs of the end user will be met; 2) using efficient, flexible, and robust practices to establish confidence in NAMs and reducing reliance on using animal data to define NAM performance; and 3) encouraging the adoption and use of NAMs by federal agencies and regulated industries. A list of NAMs accepted by US agencies can be found on the website of the US National Toxicology Program (NTP) Interagency Center for the Evaluation of Alternative Toxicological Methods (NICEATM), which supports ICCVAM’s work (NICEATM, 2021).

#### 2.2.2 Canada

The PMRA’s 2016–2021 strategic plan notes how rapidly the regulatory environment is evolving through innovations in science and puts an onus on the Agency to evolve accordingly (HC, 2016d). The strategic plan includes drivers for evolution, the importance of public confidence, the vision, mission, and key principles of scientific excellence, innovation, openness and transparency, and organizational and workforce excellence. The plan further mentions strategic enablers, which include building upon PMRA’s success in establishing and maintaining effective partnerships with provinces, territories, and other stakeholders both domestically and internationally.

#### 2.2.3 European Union

The EU is a political and economic union of 27 European countries (Member States) and its operation is guaranteed through various legal instruments. Unlike regulations and decisions that apply automatically and uniformly to all countries as soon as they enter into force, directives require Member States to achieve a certain result by transposing them into national law. In 2010, Directive 2010/63/EU on the protection of animals used for scientific purposes (EU, 2010) was adopted to eliminate disparities between laws, regulations, and administrative provisions of the Member States regarding the protection of animals used for experimental and other scientific purposes. Article 4 states that “wherever possible, a scientifically satisfactory method or testing strategy, not entailing the use of live animals, shall be used instead of a procedure,” which applies to all research purposes including regulatory toxicity testing (EU, 2010). Further, the directive lays the foundation for retrospective analyses of animal experiments, mutual acceptance of data, as well as the European Commission and Member States’ contribution to the development and validation of NAMs.

In October 2020, the EU Chemicals Strategy for Sustainability (CSS) Towards a Toxic-Free Environment was published (EC, 2020). It identified a need to innovate safety testing and chemical risk assessment to reduce dependency on animal testing while improving the quality, efficiency, and speed of chemical hazard and risk assessments. However, fulfilling its additional information requirements will more likely lead to an increase in animals used. Also, it is currently unknown whether the implementation of the CSS will open opportunities for the application of more NAMs.

## 3 Industrial chemicals

### 3.1 United States

In the US, industrial chemicals are subject to regulation under the Toxic Substances Control Act (TSCA). TSCA was originally signed into law (15 US Code [USC] §2601 *et seq.*) on 11 October 1976 with the intent “[t]o regulate commerce and protect human health and the environment by requiring testing and necessary use restrictions on certain chemical substances, and for other purposes” (Pub. L. 94-469, Oct. 11, 1976). TSCA was significantly amended in 2016 (Pub. L. 114-182, 22 June 2016). EPA is responsible for implementing and administering TSCA (see 15 USC §2601(c)) and OPPT, within EPA’s OCSPP, carries out much of that work.

TSCA provides EPA the authority to regulate new and existing chemical substances under Sections 5 and 6 of TSCA, respectively. Existing chemical substances are those on the TSCA Inventory, either those that were in commerce prior to the enactment of TSCA and grandfathered in, or those that OPPT evaluated as new chemical substances and were subsequently introduced into commerce. Entities that wish to introduce a new chemical substance or an existing chemical substance with a significant new use into commerce must submit a notification to OPPT (*i.e.*, pre-manufacture notice (PMN) or significant new use notice (SNUN)) or an appropriate exemption application, where an application is required for the exemption (e.g., low volume exemption), prior to manufacturing, including importing, the chemical substance.

Prior to the 2016 Amendments, when entities submitted a new chemical notification, no specific action by EPA was required. If EPA did not take regulatory action on the new chemical substance, the entity was allowed to manufacture the chemical substance at the expiration of the applicable review period (e.g., 90 days for a PMN). For existing chemicals, much of EPA’s TSCA activity was focused on data collection, including through section 8 rules and issuing test rules on chemical substances, including those identified by EPA’s interagency testing committee (ITC). The ITC was established under Section 4(e) of TSCA and was charged with identifying and recommending to the EPA Administrator chemical substances or mixtures that should be tested pursuant to Section 4(a) of TSCA to determine their hazard to human health or the environment. Although allowed by TSCA, EPA’s ability to regulate and restrict the use of existing chemical substances under Section 6 of TSCA was significantly impaired following a 1991 ruling by the US Court of Appeals for the Fifth Circuit (*Corrosion Proof Fittings vs. EPA*, 974 F.2d 1201), which vacated much of EPA’s TSCA Section 6 rule to ban asbestos, a rule that EPA had first announced as an advanced notice of proposed rulemaking in 1979.

The above issues with TSCA—namely new chemical substances being automatically introduced into commerce if the “clock ran out” and EPA’s limited regulation of existing chemical substances under Section 6 of TSCA—garnered Congressional attention, which culminated on 22 June 2016. On that date, then-President Obama signed the Frank R. Lautenberg Chemical Safety for the 21st Century Act into law, thereby amending TSCA (Pub.L. 114-182, 2016). The TSCA amendments placed new requirements on EPA, including requirements to review and publish risk determinations on new chemical substances, prioritize existing chemical substances as either high- or low-priority substances, and perform risk evaluations on those chemical substances identified as high-priority substances. The TSCA amendments also included new requirements for EPA to comply with specific scientific standards for best available science and weight of the scientific evidence (WoE) under Sections 26(h)-(i) of TSCA when carrying out Sections 4, 5, and 6; a new requirement to reduce testing on vertebrate animals under Section 4(h) of TSCA; and a provision giving EPA the authority to require testing on existing chemical substances by order, rather than by rule,^1^ under Section 4(a)(1) and (2) of TSCA.

The discussion that follows is focused on EPA’s authority under Section 4(h) to reduce testing on vertebrate animals, EPA’s use of this authority for new and existing chemical substances, and voluntary initiatives by the regulated community that have advanced the understanding and use of NAMs.

#### 3.1.1 General requirements

TSCA does not contain upfront vertebrate toxicity testing requirements, which allows flexibility for the adoption of NAMs. Since the enactment of the TSCA amendments, EPA has used its authority to order testing on existing chemical substances, while meeting its requirements under Section 4(h) of TSCA (EPA, 2022c). Section 4(h) includes three primary provisions: (1) the aforementioned general requirements placed on EPA for reducing and replacing the use of vertebrate animals; (2) the requirements on EPA to promote the development and incorporation of alternative testing methods, including through the development of a strategic plan and a (non-exhaustive) list of NAMs identified by the EPA Administrator; and (3) the requirements on the regulated community to consider non-vertebrate testing methods when performing voluntary testing when EPA has identified an alternative test method or strategy to develop such information.

#### 3.1.2 Regulatory flexibility

There are several sections of TSCA and the implementing regulations where EPA may use NAMs for informing its science and risk management decisions under TSCA. Data generated using NAMs may trigger reporting requirements on the regulated community. For example, under Section 8(e) of TSCA, it is possible that results generated using NAMs would trigger a reporting obligation for substantial risk for instance, if the data meet the requirements under one of EPA’s policies, such as *in vitro* skin sensitization data. In its “Strategic Plan to Promote the Development and Implementation of Alternative Test Methods Within the TSCA Program,” OPPT lists criteria that provide a starting point for considering the scientific reliability and relevance of NAMs (EPA, 2018d); however, it has yet to issue official guidance to the regulated community on its interpretation of the criteria for accepting NAMs, as meeting the scientific standards under Section 26(h) of TSCA. In addition, while OPPT has yet to issue official guidance on the criteria it uses to identify NAMs for inclusion on the list of methods approved by the EPA Administrator, the agency has presented a proposed nomination form, which provides some insight on EPA’s considerations (Simmons and Scarano, 2020).

#### 3.1.3 Implementation of NAMs

OPPT’s activities to implement NAMs have included issuing a “Strategic Plan to Promote the Development and Implementation of Alternative Test Methods Within the TSCA Program” (EPA, 2018d), establishing a list of approved NAMs (EPA, 2018c; EPA, 2019b; EPA, 2021d), and developing a draft policy allowing the use of NAMs for evaluating skin sensitization (EPA, 2018b). The latter is based on EPA’s participation in the development of the OECD guideline for Defined Approaches on Skin Sensitisation (OECD, 2021a). EPA has also performed significant outreach and collaboration to advance its understanding of NAMs, as well as educate the interested community about these technologies.

In March 2022, OPPT and ORD presented the TSCA new chemicals collaborative research effort for public comments (EPA, 2022b). This multi-year research action plan to bring innovative science to the review of new chemicals under TSCA includes: 1) refining chemical categories for read-across; 2) developing and expanding databases containing TSCA chemical information; 3) developing and refining Quantitative Structure-Activity Relationship (QSAR) and other predictive models; 4) exploring ways to apply NAMs in risk assessment; and 5) developing a decision support tool that will transparently integrate all data streams into a final risk assessment.

##### 3.1.3.1 Examples of NAM application

Already prior to the 2016 amendments to TSCA, EPA had established numerous methods for assessing chemical substances. For example, EPA has been using structure-activity relationships (SAR) for assessing the potential of new chemical substances to cause harm to aquatic organisms and an expert system to estimate potential for carcinogenicity since the 1980s (EPA, 1994).

In early 2021, OPPT issued test orders on nine existing chemical substances (EPA, 2022c). For each of the substances, OPPT ordered dermal absorption testing using an *in vitro* method validated by the OECD (OECD, 2004) instead of animal testing. After consideration of existing scientific information, EPA determined that the *in vitro* method, which is included on its list of NAMs, could be used. While EPA required the *in vitro* testing on both human and animal skin, a report has since been published analyzing 30 agrochemical formulations, which supports the use of *in vitro* assays using human skin for human health risk assessment because they are as or more protective and are directly relevant to the species of interest (Allen et al., 2021; EPA, 2021f). In reviewing test plans or test data provided to be considered *in lieu* of the ordered testing, EPA consulted with the authors of Allen et al. (2021) and subsequently determined that it would be acceptable for the *in vitro* testing to be conducted on human skin only for the chemicals subject to these particular orders.

The interested community has also been actively developing robust NAMs that can be used for regulatory decision making. For example, an entity performed voluntary *in chemico* testing on a polymeric substance that OPPT had identified as a potential hazard. The substance was classified as a poorly soluble, low-toxicity substance that, if inhaled, may lead to adverse effects stemming from lung overload. OPPT issued a significant new use rule (SNUR) on this substance, which required any entity to notify EPA (submission of a SNUN) if the polymer is manufactured, processed, or used as a respirable particle (i.e., <10 μm) (EPA, 2019c). The SNUR listed potentially useful information for inclusion in a SNUN, which consisted of a 90-day subchronic inhalation toxicity study in rats. However, the entity voluntarily undertook an *in chemico* test *in lieu* of the *in vivo* toxicity study. The *in chemico* test showed the daily dissolution rate of the polymer in simulated epithelial lung fluid exceeded the anticipated daily exposure concentrations and was, therefore, not a hazard concern from lung overload. After evaluating these data, OPPT agreed with the results and issued a final rule revoking the SNUR (EPA, 2020h). These data were subsequently published in the peer-reviewed literature (Ladics et al., 2021).

#### 3.1.4 Consumer products

In addition to the regulation of individual chemical ingredients of household products under TSCA, the Federal Hazardous Substances Act (FHSA) requires appropriate cautionary labeling on certain hazardous household products to alert consumers to the potential hazard(s) that the products may present (15 USC §1261 *et seq.*). However, the FHSA does not require manufacturers to perform any specific toxicological tests to assess potential hazards (e.g*.*, systemic toxicity, corrosivity, sensitization, or irritation). CPSC has the authority with administering FHSA. It issued guidance on the use of NAMs in 2021 (CPSC, 2022). This document lays out what factors CPSC staff will use when evaluating NAMs, IATA, and any submitted data being used to support FHSA labeling determinations. CPSCS, 2012 Animal Testing Policy (16 Code of Federal Regulations [CFR] Part 1500) strongly encourages manufacturers to find alternatives to animal testing for assessing household products.

### 3.2 Canada

The *Canadian Environmental Protection Act* (CEPA, Statutes of Canada [SC] 1999, c.33) provides the legislative framework for industrial substances, including new chemical substances and those that are currently on the Canadian market (i.e., existing substances on the Domestic Substances List [DSL]), for the protection of the environment, for the well-being of Canadians, and to contribute to sustainable development. The Safe Environments Directorate in the HECSB of Health Canada and Environment and Climate Change Canada are jointly responsible for the regulation of industrial substances under the authority of CEPA.

Existing and new substances have different legal requirements under CEPA. Accordingly, based on respective program areas, the requirements for and use of traditional and NAMs data are considered in various decision contexts including screening, prioritization, and informing risk assessment decisions. Risk assessments consider various types and sources of information, as required or available for new or existing substances respectively, including physico-chemical properties, inherent hazard, biological characteristics, release scenarios, and routes of exposure to determine whether a substance is or may become harmful according to the criteria set out in section 64 of CEPA.

The Chemicals Management Plan (CMP) was introduced in 2006 to, in part, strengthen the integration of chemicals management programs across the Government of Canada (HC, 2022e). Key elements of the CMP have been addressing the priority existing chemicals from the DSL identified through Categorization for risk assessment pursuant to obligations under CEPA and the parallel pre-market assessments of new substances not on the DSL and notified through the *New Substances Notification Regulations* provisions made under CEPA.

Under the Existing Substances Risk Assessment Program (ESRAP), the approximate 4,300 priority substances were assessed over three phases (2006–2021), requiring the development of novel methodologies and assessment strategies to address data needs as the program evolved from a chemical-by-chemical approach to the assessment of groups and classes of chemicals (HC, 2021b). The limited empirical toxicity data available for many of the priority substances necessitated the implementation of fit-for-purpose approaches, including the use of computational tools and read-across. Further, the use of streamlined approaches (HC, 2018) assisted the program to more efficiently address substances considered to be of low concern. Building on experiences and achievements from the CMP to date, the Government of Canada continues to expand on the vision for modernization. This shift takes into consideration new scientific information regarding chemicals to support innovative strategies for priority setting and to maintain a flexible, adaptive and fit-for-purpose approach to risk assessment to manage increasingly diverse and complex substances and mixtures (HC, 2021b; Bhuller et al., 2021).

The New Substances Program (NSP) is responsible for administering the *New Substances Notification Regulations* (NSNR, Statutory Orders and Regulations [SOR]/205-247 and SOR/2005-248) of CEPA (HC, 2022f). These regulations ensure that new substances (chemicals, polymers, biochemical, biopolymers, or living organisms) are not introduced into Canada before undergoing ecological and human health risk assessments, and that any appropriate or required control measures have been taken.

#### 3.2.1 General requirements

Risk assessments conducted under CEPA use a WoE approach while also applying the precautionary principle. For existing substances on the DSL, there are no prescribed data requirements to inform the assessment of a substance to determine whether it is toxic or capable of becoming toxic as defined under Section 64 of CEPA. As such, an essential first step in the risk assessment process is the collection and review of a wide range of hazard and exposure information on each substance or group of substances from a variety of published and unpublished sources, stakeholders, and various databases (HC, 2022d).

The NSNR (Chemicals and Polymers) require information be submitted in a New Substances Notification (NSN) prior to import or manufacture of a new chemical, polymer, biochemical, or biopolymer in Canada. The NSNR (Chemicals and Polymers) also require that a notifier submit all other relevant data in their possession relevant to the assessment. Subsection 15(1) of the NSNR (Chemicals and Polymers) states that conditions and procedures used must be consistent with conditions and procedures set out in the OECD TG that are current at the time the test data are developed, and should comply with GLP.

Information in support of a NSN may be obtained from alternative test protocols, WoE, read-across, as well as from (Q)SARs [calculation or estimation methods (e.g., EPI Suite)]. The NSP may use various NAMs in their risk assessment, and may accept (and has accepted) test data which use NAMs, as discussed in further detail below.

#### 3.2.2 Regulatory flexibility

For existing substances on the DSL under CEPA, there are no set submission requirements prior to an assessment, which inherently presents the need for flexibility and the opportunity to integrate novel approaches. NAM data are often used to support the assessment of the potential for risk from data poor substances. Since these data poor substances are unlikely to have required or available guideline studies, NAMs, including computational modelling, *in vitro* assays, QSAR and read-across, are used as approaches to address data needs offering an opportunity for a risk-based assessment where this may have been challenging in the past (HC, 2022a). For new substances, the NSP supports ongoing NAM development, as well as monitoring studies, to provide information on levels of substances of interest in the environment; both are used to fill risk assessment data gaps. In 2021, the NSP published a draft updated Guidance Document for the Notification and Testing of New Substances: Chemicals and Polymers (HC, 2021c). Section 8.4 of this Guidance Document lists examples of accepted test methods, which could in the future include NAMs as they are shown to be scientifically valid. Under the NSNR, alternative approaches will be acceptable when, in the opinion of the NSP, they are determined to provide a scientifically valid measure of the endpoint under investigation that is deemed sufficient for the purposes of the risk assessment. NAM data are evaluated on a case-by-case basis and can form part of the WoE of an assessment.

#### 3.2.3 Implementation of NAMs

Given the paucity of data available for many substances on the market, as well as for new substances, there is a long history of using alternative approaches for hazard identification and characterization in support of new and existing substances risk assessment decisions. Over the last 2 decades, a variety of NAMs have been used by different program areas to address information gaps for risk assessment. The approaches implemented have been fit-for-purpose and largely determined by the data need, the timeline, the type of chemical(s), and the level of complexity associated with the assessment (HC, 2016a). Most notably for existing substances, *in silico* models, (Q)SAR, and read-across have been the most widely used methods with the progressive adoption and expanded use of computational toxicology and automated approaches ongoing for both ESRAP and the NSP. More specific details on the evolution of the ESRAP under CEPA are highlighted in the CMP Science Committee meeting report (HC, 2021b).

There are currently no formal criteria that have been published in order to achieve regulatory acceptance for the implementation of NAMs for existing substances in Canada. However, experience and efficiencies have been gained through the strategic development and implementation of streamlined risk-based approaches that support rapid and robust decision-making. To this end, a number of science approach documents (SciAD) have been published describing and demonstrating the implementation of NAMs to evaluate the potential for environmental or human health risk from industrial substances (HC, 2022c). SciADs are published under section 68 of CEPA, and do not include regulatory conclusions; however, the approach and results described within a SciAD may form the basis for a risk assessment conclusion when used in conjunction with any other relevant and available information. Furthermore, the implementation of NAMs as described in SciADs can also be used to support the identification of priorities for data gathering, data generation, further scoping, and risk assessment (HC, 2022c).

In advancing the vision for progressive chemicals management programs, which includes reduced use of animals and integration of NAMs, it is recognized that there is an ongoing need to develop flexible, adaptive, and innovative approaches. Accordingly, the ESRAP continues to expand the use of computational and *in vitro* models as well as evidence integration strategies to identify and address emerging priority substances. Key to successful implementation moving forward are the productive partnerships with the international regulatory and research communities to continue to build confidence and harmonization for the use of alternative test methods and strategies in chemical risk assessment (Krewski et al., 2020; Bhuller et al., 2021).

Data generated using NAMs may be accepted to fulfil any of the NSNR’s test data requirements for an NSN when, in the opinion of the NSP, such data are determined to provide a scientifically valid measure of the endpoint under investigation that is deemed sufficient for the purposes of the risk assessment. The NSP will assess if the method has been satisfactorily validated in terms of scientific rigor, reproducibility, and predictability. Guidance is provided to notifiers who wish to submit information using NAMs during Pre-Notification Consultation meetings with NSP staff, or notifiers can consult Sections 5.4 and 8.4 of the respective Guidance Document (HC, 2021c). Alternative methods that may be accepted by the NSP to meet NSNR requirements include any internationally recognized and accepted test methods (e.g., *in vitro* skin irritation, gene mutation, and chromosomal aberration). Data such as (Q)SAR, read-across (greater than 80% structural similarity), and WoE may be accepted on a case-by-case basis.

##### 3.2.3.1 Examples of NAM applications

As noted above, beyond the use of *in silico* models and read-across, examples of NAM applications for existing substances have been published as SciADs outlining NAM-based methods for prioritization and assessment (HC, 2022c). Specifically, the SciAD “Threshold of Toxicological Concern (TTC)-based Approach for Certain Substances” has been applied to evaluate a subset of existing substances on the DSL identified as priorities for assessment under subsection 73(1) of CEPA and/or were considered a priority based on human health concerns (HC, 2016c). More recently, the SciAD “Bioactivity exposure ratio: Application in priority setting and risk assessment approach” was developed outlining a quantitative risk-based approach to identify substances of greater potential concern or substances of low concern for human health (HC, 2021f). This proposed approach for NAM application builds on a broad retrospective analysis under the APCRA (Paul Friedman et al., 2020) and considers high-throughput *in vitro* bioactivity together with high-throughput toxicokinetic modelling to derive an *in vitro*-based point of departure. As technologies continue to advance and additional sources of data from NAMs emerge, these may also be considered in the ongoing expansion of the approach to support the derivation of molecular-based PODs as part of a tiered testing scheme. Further work is underway to build approaches for the interpretation of transcriptomics data and to enhance the use of QSAR and machine learning to enrich evidence integration and WoE evaluation using IATA frameworks across toxicological endpoints of regulatory relevance.

New substances are inherently data-poor substances and, as a result, the NSP typically accepts a variety of alternative approaches and NAM data to meet data requirements under the NSNR. QSAR data and read-across data using analogues have historically been used to meet data requirements under the NSNR, particularly for physico-chemical data requirements or in combination with other data to provide a WoE for toxicity data. More recently, newly validated *in vitro* methods for skin irritation and skin sensitization (OECD, 2021a) have been accepted to meet data requirements under the NSNR. The NSP participates in active research programs to develop NAMs for complex endpoints, such as genotoxicity and systemic toxicity. Although not a regulatory requirement, *in vitro* eye irritation tests are also frequently received by the NSP.

### 3.3 European Union

In 2006, a significant number of updates and revisions were introduced into the EU chemicals policy with the introduction of Regulation (EC) No 1907/2006 concerning the Registration, Evaluation, Authorisation and Restriction of Chemicals (REACH) (EC, 2006). REACH entered into force on 1 June 2007, and introduced a single system for the regulation of chemicals, transferring the burden of proof concerning the risk assessment of substances from public authorities to companies. The purpose of REACH, according to Article 1(1), is to “ensure a high level of protection of human health and the environment, including the promotion of alternative methods for assessment of hazards of substances, as well as the free circulation of substances on the internal market while enhancing competitiveness and innovation” (EC, 2006). The Regulation established ECHA to manage and implement the technical, scientific, and administrative aspects of REACH. Enforcement of REACH is each EU Member State’s responsibility and, therefore, ECHA has no direct enforcement responsibilities (ECHA, n.d.a). In addition to REACH, Regulation (EC) 1272/2008 on classification, labelling and packaging of substances and mixtures (CLP regulation) (EC, 2008b) was introduced to align the EU chemical hazard labeling system with the United Nations Economic Commission for Europe (UNECE)’s Globally Harmonised System of Classification and Labelling of Chemicals (GHS). Both REACH and CLP regulation are currently undergoing extensive revisions at the time of submission of this manuscript.

#### 3.3.1 General requirements

REACH applies to all chemical substances; however, certain substances that are regulated by other legislations (*e.g.*, biocides, PPPs, or medical drugs) may be (partially) exempted from specific requirements (ECHA, n.d.f). Substances used in cosmetic products remain a contentious issue with them being subject to an animal testing ban under the EU regulation on cosmetics products (EC, 2009b), yet ECHA continues to request new *in vivo* testing under certain circumstances such as for risk assessment for worker exposure (ECHA, 2014). The interplay between the two regulations is under review by the European Court of Justice (‘Symrise v ECHA’ (2021), T655/20, ECLI:EU:T:2021:98 and ‘Symrise v ECHA' (2021), T-656/20, ECLI:EU:T:2021:99).

Whilst REACH is not a pre-marketing approval process in the strictest sense of the definition, it works on the principle of no data, no market with responsibility placed on registrants to manage the risks from chemicals and to provide safety information on the substances. Thus, companies bear the burden of proof to identify and manage the risks linked to the substances they manufacture or import and place on the market in the EU. They must demonstrate how the substance can be safely used and must communicate the risk management measures to the users. Companies must register the chemical substances they manufacture or import into the EU at more than one tonne per year with ECHA. The registration requirement under REACH “applies to substances on their own, in mixtures, or, in certain cases, in articles” (ECHA, n.d.c). Registration is governed by the “one substance, one registration” principle, where manufacturers and importers of the same substance must submit their registration jointly. Companies must collect information on the properties and uses of their substances and must assess both the hazards and potential risks presented by these substances. The companies compile all of this information in a registration dossier and submit it to ECHA. The standard information requirements for the registration dossier depends on the tonnage band of the chemical substance (ECHA, n.d.b). The information required is specified in Annexes VI to X of REACH and include physico-chemical data, toxicology information, and ecotoxicological information.

ECHA receives and evaluates individual registrations for their compliance (ECHA, n.d.f). EU Member States evaluate certain substances to clarify initial concerns for human health or for the environment. ECHA’s scientific committees assess whether any identified risks from a hazardous substance are manageable, or whether that substance must be banned. Before imposing a ban, authorities can also decide to restrict the use of a substance or make it subject to a prior authorization.

The CLP regulation requires that relevant information on the characteristics of a substance, classification of toxicity endpoints, and pertinent labelling of a substance or substances in mixtures be notified to ECHA when placed on the EU market (EC, 2008b). In this way, the toxicity classification and labeling of the substance are harmonized both for chemical hazard assessment and consumer risk. In cases where there are significant divergences of scientific opinion, further review of scientific data can proceed (EC, 2008b). New testing is normally not requested for CLP purposes alone unless all other means of generating information have been exhausted and data of adequate reliability and quality are not available (ECHA, n.d.e).

The discussion that follows is focused on the EU’s efforts under REACH to reduce testing on vertebrate animals to assess human health effects. This concept lies at the very foundation of REACH, which states in the second sentence of the Preamble that it should “promote the development of alternative methods for the assessment of hazards of substances” (EC, 2006).

#### 3.3.2 Regulatory flexibility

According to Article 13(1) of REACH, “for human toxicity, information shall be generated whenever possible by means other than vertebrate animal tests, through the use of alternative methods, for example, in vitro methods or qualitative or quantitative structure-activity relationship models or from information from structurally related substances (grouping or read-across)” (EC, 2006). Further, according to Article 13(2), the European Commission may propose amendments to the REACH Annexes and the Commission Regulation, which lists approved test methods (EC, 2008a), to “replace, reduce or refine animal testing.” Under Title III of REACH, on Data Sharing and Avoidance of Unnecessary Testing, Article 25(1) requires that testing on vertebrate animals must be undertaken only as a last resort; however, the interpretation of Articles 13 and 25 of REACH are often matters of dispute in European Court of Justice (‘Federal Republic of Germany v Esso Raffinage’ (2021), C-471/18 P, ECLI:EU:C:2021:48), ECHA Board of Appeal (*e.g.*, cases A-005-2011 and A-001–2014), and European Ombudsman cases (cases 1568/2012/(FOR)AN, 1606/2013/AN and 1130/2016/JAS).

In addition, to reduce animal testing and duplication of tests, study results from tests involving vertebrate animals should be shared between registrants (EC, 2006). Furthermore, where a substance has been registered within the last 12 years, a potential new registrant must, according to Article 27, request from the previous registrant all information relating to vertebrate animal testing that is required for registration of the substance. Before the deadline to register all existing chemicals by 31 May 2018, companies (i.e., manufacturers, importers, or data owners) registering the same substance were legally required to form substance information exchange fora (SIEFs) to help exchange data and avoid duplication of testing for existing chemicals (EC, 2006).

REACH standard information requirements for registration dossiers contain upfront testing requirements on vertebrate animals with some flexibility to allow the use of NAMs. Registrants are encouraged to collect all relevant available information on the substance, including any existing data (human, animal, or NAMs), (Q)SAR predictions, information generated with analogue chemicals (read-across), and *in chemico* and *in vitro* tests. In addition, REACH foresees that generating information required in Annexes VII-X may sometimes not be necessary or possible. In such cases, the standard information for the endpoint may be waived. Criteria for waiving are outlined in Column 2 of Annexes VII-X, while criteria for adapting standard information requirements are described in Annex XI of REACH (ECHA, 2016a). In addition to the use of OECD test guidelines, data from *in vitro* methods that meet internationally agreed pre-validation criteria as defined in OECD GD 34 are considered suitable for use under REACH when the results from these tests indicate a certain dangerous property. However, negative results obtained with pre-validated methods have to be confirmed with the relevant *in vivo* tests specified in the Annexes. Whether the aforementioned current revision of REACH and CLP regulations will bring about opportunities to include more NAMs in the assessment of industrial chemicals or lead to an increase in animal testing is to be seen.

#### 3.3.3 Implementation of NAMs

The REACH annexes were amended in 2016 and 2017 to require companies to use NAMs for certain endpoints under certain conditions. Following these amendments, the use of non-animal tests have tripled for skin corrosion/irritation, quadrupled for serious eye damage/eye irritation, and increased more than 20-fold for skin sensitization (ECHA, 2020).

REACH requires that robust study summaries be published on the ECHA website. This helps registrants identify additional data for their registrations and facilitates the identification of similar or identical substances (ECHA, 2020). ECHA’s public chemical database may also be used to conduct retrospective data analyses and other research efforts, when the level of detailed data needed are present in such reports (Luechtefeld et al., 2016a; Luechtefeld et al., 2016b; Luechtefeld et al., 2016c; Luechtefeld et al., 2018; Knight et al., 2021).

ECHA engages in OECD expert groups and reviews test guidelines for both animal and non-animal methods. For example, ECHA contributed to the *in vitro* OECD test guidelines for skin and eye irritation in 2016 and skin sensitization in 2017. In addition, ECHA was involved in the finalization of the OECD “Defined Approaches on Skin Sensitisation Test Guideline” (OECD, 2021a). In October 2021, ECHA published advice on how REACH registrants can use the defined approaches guideline, and this was the first official guidance outlining how to use *in silico* tools, such as the QSAR Toolbox, to assess skin sensitization (ECHA, 2021). Furthermore, ECHA also engages in largescale European research projects (e.g., EU-ToxRisk) where they review mock dossiers based on NAMs that have been developed in these projects.

Before registrants conduct higher-tier tests for assessing the safety of chemicals they import or manufacture, Article 40 of REACH requires that they submit details on their testing plans to ECHA (ECHA, n.d.d). In that submission, companies must detail how they considered NAMs before proposing an animal test. ECHA must agree on these proposals before a company can conduct a new animal test under Annex IX or X. ECHA may reject, accept, or modify the proposed test. As required by REACH, all testing proposals involving testing on vertebrate animals are published on ECHA’s website to allow citizens and organizations the opportunity to provide information and studies about the substance in question (ECHA, n.d.d). ECHA will inform the company that submitted the testing proposal of the Member State Committee’s decision and is required to take into account all studies and scientifically valid information submitted as part of the third-party consultation when making its decision.

##### 3.3.3.1 Examples of NAM application

The most commonly used NAM under REACH is the read-across approach, where relevant information from analogous substances is used to predict the properties of target substances (ECHA, 2020). Before read-across is accepted by ECHA, it must be justified by the registrant and, therefore, to facilitate its use, ECHA developed a read-across assessment framework (ECHA, 2017). Additionally, ECHA is holding different expert meetings with stakeholders including industry representatives and NGOs to enhance and combine knowledge and to avoid overlap and duplication. Thus, ECHA encourages companies to avoid duplicate animal tests and share any data they have on their substance if requested by a registrant of an analogous substance. For example, based on *in vitro* ToxTracker assay results and read-across data from the analogue substance aminoethylpiperazine, ECHA has not requested *in vivo* genotoxicity data for N,N,4-trimethylpiperazine-1-ethylamine, which was registered by two companies in a joint submission (ECHA, 2019)*.*

## 4 Pesticides and plant protection products

### 4.1 United States

The Federal Insecticide, Fungicide, and Rodenticide Act (FIFRA; 7 USC §136) requires all pesticides sold or distributed in the US to be registered with the EPA, unless otherwise exempted. EPA then has authority under the Federal Food, Drug, and Cosmetic Act (FD&C Act; 21 USC §301 *et seq.*) to set the maximum amount of pesticide residues permitted to remain in/on food commodities or animal feed, which are referred to as tolerances. In 1996, both of these statutes were amended by the Food Quality Protection Act (FQPA), which placed new requirements on EPA, including making safety findings (i.e., “a reasonable certainty of no harm”) when setting tolerances (Pub.L. 104-170, 1996).

OPP, within EPA’s OCSPP, has the delegated authority with administering the above laws and is responsible for pesticide evaluation and registration. This includes registration of new pesticide active ingredients and products, as well as new uses for currently registered pesticides. Additionally, OPP reviews each registered pesticide at least every 15 years as part of the Registration Review process to determine whether it continues to meet registration standards. A pesticide product may not be registered unless the EPA determines that the pesticide product will not cause unreasonable adverse effects on the environment (as defined by 7 USC §136(bb)).

#### 4.1.1 General requirements

Data requirements for pesticide registration are dependent on the type of pesticide (i.e., conventional, biopesticide, or antimicrobial) and use pattern (e.g., food versus non-food, or anticipated routes of exposure) and are laid out in 40 CFR Part 158. Unlike TSCA, FIFRA and its implementing regulations require substantial upfront testing to register a pesticide in the US, such as product chemistry data to assess labeling, product performance data to support claims of efficacy, studies to evaluate potential hazards to humans, studies to evaluate potential hazards to non-target organisms, environmental fate data, and residue chemistry and exposure studies to determine the nature and magnitude of residues. The data are used to conduct comprehensive risk assessments to determine whether a pesticide meets the standard for registration.

#### 4.1.2 Regulatory flexibility

US regulations give EPA substantial discretion to make registration decisions based on data that the Agency deems most relevant and important for each action. As stated in the CFR, under Section 158.30, the studies and data required may be modified on an individual basis to fully characterize the use and properties of specific pesticide products under review. Also, the data requirements may not always be considered appropriate. For instance, the properties of a chemical or an atypical use pattern could make it impossible to generate the required data or the data may not be considered useful for the evaluation. As a result, Section 158.45 permits OPP to waive data requirements as long as there are sufficient data to make the determinations required by the applicable statutory standards.

To assist staff in focusing on the most relevant information and data for assessment of individual pesticides, OPP published “Guiding Principles for Data Requirements” (EPA, 2013a). The document describes how to use existing information about a pesticide to identify critical data needs for the risk assessment, while avoiding generation of data that will not materially influence a pesticide’s risk profile and ensuring there is sufficient information to support scientifically sound decisions. When data from animal testing will not contribute to decision making, OPP has developed processes to waive guideline studies and/or apply existing toxicological data for similar substances (i.e., bridging). Detailed guidance on the scientific information needed to support a waiver or bridging justification has been developed by OPP for acute (EPA, 2012; EPA, 2016a; EPA, 2020c) and repeat dose (EPA, 2013b) mammalian studies.

Interdivisional expert committees within OPP are tasked with considering waiver requests on a case-by-case basis. The Hazard and Science Policy Council (HASPOC) is tasked with evaluating requests to waive most guideline mammalian toxicity studies, except acute systemic lethality and irritation/sensitization studies (which are referred to as the acute six-pack). HASPOC is comprised of toxicologists and exposure scientists from divisions across OPP focused on conducting human health risk assessments and it utilizes a WoE approach described in its guidance on “Part 158 Toxicology Data Requirements: Guidance for Neurotoxicity Battery, Subchronic Inhalation, Subchronic Dermal and Immunotoxicity Studies” (EPA, 2013b). This includes consideration of multiple lines of evidence, such as physico-chemical properties, information on exposure and use pattern, toxicological profiles, pesticidal and mammalian mode of action information, and risk assessment implications. Although this guidance was developed to address particular toxicity studies, the same general WoE approach is applied by HASPOC when considering the need for other toxicity studies for pesticide regulatory purposes. Between 2012 and 2018, the most common studies requested to be waived were acute and subchronic neurotoxicity, subchronic inhalation, and immunotoxicity studies (Craig et al., 2019). For the acute six-pack studies, the Chemistry and Acute Toxicology Science Advisory Council (CATSAC) was formed to consider bridging proposals and/or waivers using the aforementioned waiving and bridging guidance documents. For example, following a retrospective analysis, the agency released guidance for waiving acute dermal toxicity tests (US EPA, OCSPP, and OPP, 2016). The progress of HASPOC and CATSAC is continuously tracked and reported on an annual basis (Craig et al., 2019; EPA, 2020a, 2021b).

Beyond waiving studies that do not contribute to regulatory decision making, OPP has the ability to use relevant NAMs to replace, reduce, and refine animal studies. The CFR provides OPP with considerable flexibility under Section 158.75 to request additional data beyond the Part 158 data requirements that may be important to the risk management decision. NAMs can be considered and accepted for these additional data, when appropriate.

#### 4.1.3 Implementation of NAMs

Several documents describe OPP’s strategies to reduce reliance on animal testing and incorporate relevant NAMs. For example, in addition to overarching EPA strategic plans (see Section 2.1.2.1.), OPP consulted the FIFRA Scientific Advisory Panel (SAP) on strategies and initial efforts to incorporate molecular science and emerging *in silico* and *in vitro* technologies into an enhanced IATA (EPA, 2011). The long-term goal identified for this consultation was a transition from a paradigm that requires extensive *in vivo* testing to a hypothesis-driven paradigm where NAMs play a larger role.

Unlike TSCA that requires OPPT to maintain a (non-exhaustive) list of NAMs that are accepted, OPP does not have a similar statutory requirement. However, OPP does maintain a website with strategies for reducing and replacing animal testing based on studies and approaches that are scientifically sound and supportable (EPA, 2022a). For many of these strategies, OPP has worked closely with other EPA offices, including OPPT and ORD, to develop and implement plans and tools that advance NAMs. Additionally, OPP works with a wide range of external organizations and stakeholders, including other US federal agencies, international regulatory agencies, animal protection groups, and pesticide registrants.

These collaborations have resulted in several agency documents for specific NAM applications. As mentioned in previous sections, there have been national and international efforts to develop defined approaches for skin sensitization in which OPP participated, along with OPPT, PMRA, ECHA, and other stakeholders. In 2018, a draft policy document was published jointly by OPP and OPPT on the use of alternatives approaches (*in silico*, *in chemico,* and *in vitro*) that can be used to evaluate skin sensitization in lieu of animal testing with these approaches accepted as outlined in the draft policy upon its release (EPA, 2018b). As international work develops through the OECD, this policy will be updated to accept additional defined approaches as appropriate. OPP also has a policy on the “Use of an Alternate Testing Framework for Classification of Eye Irritation Potential of EPA Pesticide Products,” which focuses on the testing of antimicrobial cleaning products but can be applied to conventional pesticides on a case-by-case basis (EPA, 2015).

Collaborative efforts have also resulted in numerous publications in scientific journals that allow for communication of scientific advancements and analyses, while building confidence in NAM approaches that can support regulatory decisions. For example, analyses have been published demonstrating that many of the *in vitro* or *ex vivo* methods available for eye irritation are equivalent or scientifically superior to the rabbit *in vivo* test (Clippinger et al., 2021). Additionally, OPP established a pilot program to evaluate a mathematical tool (GHS Mixtures Equation) as an alternative to animal oral inhalation toxicity studies for pesticide formulations. After closing the submission period in 2019, OPP worked with NICEATM to conduct retrospective analyses, which demonstrated the utility of the GHS Mixtures Equation to predict oral toxicity, particularly for formulations with lower toxicity (Hamm et al., 2021). Furthermore, OPP participated in a project to rethink chronic toxicity and carcinogenicity assessment for agrochemicals (called “ReCAAP”). The workgroup, consisting of scientists from government, academia, non-governmental organizations, and industry stakeholders, aimed to develop a reporting framework to support a WoE safety assessment without conducting long-term rodent bioassays. In 2020, an EPA Science Advisory Board meeting was held to discuss reducing the use of animals for chronic and carcinogenicity testing, which included comment on the ReCAAP project (EPA, 2020f), and feedback from the consultation was incorporated into a published framework (Hilton et al., 2022).

##### 4.1.3.1 Examples of NAM application

OPP has recently used NAMs to derive points of departure for human health risk assessment. For isothiazolinones, which are material preservatives that are known dermal sensitizers, NAMs were utilized to support a quantitative assessment (EPA, 2020g). *In chemico* and *in vitro* assays were performed on each chemical to derive concentrations that can cause induction of skin sensitization and were used as the basis of the quantitative dermal sensitization evaluation. The NAM approaches used in the assessment have been shown to be more reliable, human-relevant, and mechanistically driven, and able to better predict human sensitizing potency when compared to the reference test method, the mouse local lymph node assay (EPA, 2020d).

In addition, as part of a registration review, a NAM approach was used to evaluate inhalation exposures for the fungicide chlorothalonil, which is a respiratory contact irritant (EPA, 2021c). The approach utilizes an *in vitro* assay to derive an inhalation point of departure in conjunction with *in silico* dosimetry modeling to calculate human equivalent concentrations for risk assessment (Corley et al., 2021; McGee Hargrove et al., 2021). The approach, which was reviewed and supported by a FIFRA Scientific Advisory Panel (EPA, 2018a), provided an opportunity to overcome challenges associated with testing respiratory irritants, while also incorporating human relevant information.

Further, OPP has been shifting its testing focus from developmental neurotoxicity (DNT) guideline studies to more targeted testing approaches. In addition to evaluating life stage sensitivity with studies based on commonly accepted modes of action, such as comparative cholinesterase assays and comparative thyroid assays, researchers from ORD have participated in an international effort over the past decade to develop a battery of NAMs for fit-for-purpose evaluation of DNT (Fritsche et al., 2017; Bal-Price et al., 2018a; Bal-Price et al., 2018b; Sachana et al., 2019). As part of this effort, ORD researchers developed *in vitro* assays using microelectrode array network formation array (MEA NFA) and high-content imaging (HCI) platforms to evaluate critical neurodevelopmental processes. Additional *in vitro* assays have been developed by researchers funded by EFSA and, together with the ORD assays, form the current DNT NAM battery. The FIFRA SAP supported the use of the data generated by the DNT NAM battery as part of a WoE for evaluating DNT potential and recognized the potential for the battery to continuously evolve as the science advances (EPA, 2020i). The OECD DNT expert group, which includes staff from OPP and ORD as well as representatives from other US agencies (e.g., NTP, FDA), is also considering several case studies on integrating the DNT battery into an IATA. Furthermore, data from the battery along with toxicokinetic assessment and available *in vivo* data were recently used in a WoE to support a DNT guideline study waiver (Dobreniecki et al., 2022).

OPP also collaborated with NICEATM to complete retrospective analyses of dermal penetration triple pack studies (Allen et al., 2021). A triple pack consists of an *in vivo* animal study and *in vitro* assays using human and animal skin and are used to derive DAFs applied to convert oral doses to dermal-equivalent doses to assess the potential risk associated with dermal exposures. The retrospective analyses demonstrated that *in vitro* studies alone provide similar or more protective estimates of dermal absorption with limited exception. The use of human skin for human health risk assessment has the added advantage of being directly relevant to the species of interest and avoiding overestimation of dermal absorption using rat models. These analyses are being used by OPP to support its consideration of results from acceptable *in vitro* studies in its WoE evaluations to determine an appropriate dermal absorption factor (DAF) for human health risk assessment on a chemical-by-chemical basis.

### 4.2 Canada

In Canada, pest control products and the corresponding technical grade active ingredient are regulated under the *Pest Control Products Act* (PCPA; SC 2002, c.28). The PCPA and its associated Regulations govern the manufacture, possession, handling, storage, transport, importation, distribution, and use of pesticides in Canada. Pesticides, as defined in the PCPA, are designed to control, destroy, attract, or repel pests, or to mitigate or prevent pests’ injurious, noxious, or troublesome effects. Therefore, these properties and characteristics that make pesticides effective for their intended purposes may pose risks to people and the environment.

PMRA is the branch of Health Canada responsible for regulating pesticides under the authority of the PCPA. Created in 1995, PMRA consolidates the resources and responsibilities for pest management regulation in Canada. PMRA’s primary mandate is to prevent unacceptable risks to Canadians and the environment from the use of these products. Section 7 of the PCPA provides the authority for PMRA to apply modern, evidence-based scientific approaches to assess whether the health and environmental risks of pesticides proposed for registration (or amendment) are acceptable, and that the products have value. Section 16 of the PCPA provides the legislative oversight for PMRA to take the same approach when regularly and systematically reviewing whether pesticides already on the Canadian market continue to meet modern scientific standards. PMRA’s guidance document “A Framework for Risk Assessments and Risk Management of Pest Control Products” provides the well-defined and internationally recognized approach to risk assessment, management, and decision-making. This framework includes insights on how interested and affected parties are involved in the decision-making process. It also describes the components of the risk (health and environment) and value assessments. For example, the value assessment’s primary consideration is whether the product is efficacious. In addition, this assessment contributes to the establishment of the use conditions required to assess human health and environment risks (HC, 2021a).

#### 4.2.1 General requirements

In Canada, many pest control products are categorized as conventional chemicals, and include insecticides, fungicides, herbicides, antimicrobials, personal insect repellents, and certain companion animal products such as spot-on pesticides for flea and tick control. Non-conventional chemicals, such as biopesticides (e.g., microbial pest control agents) and essential oil-based personal insect repellents, are also regulated under the PCPA.

The scope of the information provided in this section is most applicable to the health component of the risk assessment for domestic registrations of conventional chemicals (the end-use product and active ingredient). The information provided hereafter excludes the value and environment components along with products, such as food items (e.g., table salt), which are of interest to the organic growers in Canada. Biopesticides and non-conventionals are also outside the scope of this paper.

PMRA relies on a system that links the data requirements (data-code or DACO tables) to proposed use-sites, which are organized using three categories: Agriculture, Industry, and Society (HC, 2006). Given that pest control products can be used on more than one use-site, these sites are further sub-categorized. For example, PMRA’s use-site category 14 is for “Terrestrial Food Crops” and includes crops grown outdoors as a source for human consumption (HC, 2013b). The system of linking DACOs with use-site categories is similar to what is used by the US EPA and internationally by the OECD (HC, 2006). The PMRA DACO tables include required (R) and conditionally required (CR) data that are tailored for each use site and take into consideration potential routes, durations, and sources of exposure to humans and the environment. It is important to note that the CR data are only required under specified conditions. In addition, PMRA will consider a request to waive any data requirement, but such waiver requests must be supported by a scientific rationale demonstrating that the data are not required to ensure the protection of human health. In particular, PMRA published a guidance document for waiving or bridging of mammalian acute toxicity tests for pesticides in 2013 (HC, 2013a). This document served as the starting point for the development and subsequent release of the 2017 OECD technical document on the same subject (OECD, 2017).

#### 4.2.2 Regulatory flexibility

The specific data requirements for the registration of pest control products in Canada are not prescribed in legislation under the PCPA. PMRA, therefore, has greater flexibility in either adopting or adapting methods under the PCPA in comparison to other jurisdictions where these data requirements are established in law. Therefore, while the PCPA provides the overarching components for the assessments (i.e., health, environment, and value) it also provides for the flexibility to use policy instruments along with guidance documents to provide the details on the data requirements to satisfy these legislative components. This approach also provides the opportunity for PMRA to engage all stakeholders through webinars, meetings, and public consultations when developing or making major changes to these documents. This open and transparent approach is aligned with PMRA’s strategic plan (HC, 2016d), which includes incorporating modern science by building scientific, regulatory, and public confidence in these approaches through collaborative processes. The ability to rely on policy instruments and guidance documents does not preclude PMRA from making regulatory changes, when necessary; however, the experience, thus far, with NAMs supports the current approach of relying on multi-stakeholder collaborations that result in the development of guidance documents, science policy notes, and/or published articles in reputable scientific journals.

#### 4.2.3 Implementation of NAMs

PMRA’s 2019-2020 annual report highlights the 25th anniversary of this branch of Health Canada while noting a major transformation initiative of the pesticide program (HC, 2021e). Building upon the strategic plan (see Section 2.2.2), the program renewal project considers the changing landscape and the need for PMRA to keep pace with this change. The 2019-2020 and 2020-2021 reports include a section on evaluating new technologies, which includes opportunities to reduce animal testing wherever possible. Specifically, the use of NAMs, including *in vitro* assays, predictive *in silico* models, mechanistic studies, and existing data for human health and environmental assessment of pesticides is noted (HC, 2021e; Hc, 2022b).

Bhuller et al. (2021) provides the first Canadian regulatory perspective on the approach and process towards the implementation of NAMs in Canada for pesticides and industrial chemicals (Bhuller et al., 2021). It acknowledges foundational elements, such as the 2012 Council of Canadian Academies (CCA, 2012) expert panel report, “Integrating Emerging Technologies into Chemical Assessment,” used to establish the overall vision. The process for identifying, exploring, and implementing NAMs emphasizes the importance of mobilizing teams and fostering a mindset that enables a regulatory pivot towards NAMs. In addition, the importance of engagement and multi-stakeholder collaboration is identified as a pillar for building regulatory, scientific, and public confidence in NAMs along with the broader acceptance of the alternative approaches.

PMRA collaborates with stakeholders on the development of NAMs and their potential implementation for regulatory purposes. For example, PMRA is collaborating with the interested community through several ongoing multi-stakeholder initiatives designed to explore NAMs, at the national and international levels (Bhuller et al., 2021). Another example is several academic-led initiatives along with research and consulting firms that are immersed in developing models, including open-source models. This includes the University of Windsor’s Canadian Centre for Alternatives to Animal Methods (CCAAM) and CaCVAM. Within Health Canada, voluntary efforts amongst regulatory and research scientists have resulted in the publication of NAM-relevant documents, such as the current Health Canada practices for using toxicogenomics data in risk assessment (HC, 2019).

##### 4.2.3.1 Examples of NAM application

Multiple NAMs and alternatives to animal testing have been co-developed, adapted, or adopted by the PMRA. Examples include the OECD defined approach for skin sensitization (OECD, 2021a), use of a WoE framework for chronic toxicity and cancer assessment (Hilton et al., 2022), and PMRA’s “Guidance for Waiving or Bridging of Mammalian Acute Toxicity Tests for Pesticides” (HC, 2013a). In addition, PMRA no longer routinely requires the acute dermal toxicity assay (HC, 2017), the one-year dog toxicity test (Linke et al., 2017; HC, 2021d), or the *in vivo* dermal absorption study (Allen et al., 2021) in alignment with the US EPA. PMRA will consider these and other NAMs *in lieu* of animal testing for specific pesticides by applying a WoE approach to ensure that the available information is sufficient and appropriate for hazard characterization and the assessment of potential human health risks.

Building upon the strategic plan and the importance of staying current with scientific advancements in an open and transparent manner, PMRA’s DACO guidance document for conventional pesticides includes a document history table that enables PMRA to demonstrate the “evergreen” nature of the DACOs while providing an overview of the changes and the corresponding rationales (HC, 2021d). For example, PMRA’s science-policy work, resulting in no longer routinely requiring the acute dermal toxicity study, is captured in this table with a reference to the science-policy document (SPN 2017-03) (HC, 2017). The latter then provides details on public consultation processes and the robust retrospective analysis that was undertaken under the auspices of the RCC (HC, 2017).

### 4.3 European Union

In the EU, the term “pesticides” includes (1) active ingredient and PPPs, which are intended for use on plants in agriculture and horticulture, and (2) biocides, which are used in non-botanical applications, such as rodenticides or termiticides. PPPs and their active ingredients are regulated under Regulation (EC) No 1107/2009 (EC, 2009a). Commission Regulation (EU) No 283/2013 lists the data requirements for active ingredients (EU, 2013c), and Commission Regulation (EU) No 284/2013 lists the data requirements for PPPs (EU, 2013d). Biocides, however, are regulated separately under Regulation (EU) No 528/2012 and are not discussed in this paper (EU, 2012). In addition, the CLP regulation (see Section 3.3) applies to both PPPs and biocides.

The EU is a diverse group of countries as it relates to food consumption, agricultural pests, weather, and level of development, thus, the risk assessment and management procedures were developed to account for the varied needs of different Member States. First, an evaluation of the active ingredient dossier is conducted by a Rapporteur Member State. Then, EFSA peer reviews the dossier evaluation. The peer reviewed risk assessment of the active ingredient is considered by the European Commission, who makes a proposal on whether to authorize the active ingredient, followed by the EU Member States, who vote on final risk management decisions. Once an active ingredient is authorized, individual Member States consider applications for approval of PPPs containing that active ingredient and propose maximum levels of pesticide residues permitted to remain in/on food commodities or animal feed. Finally, the European Commission (often with input from EFSA) will decide whether to approve those maximum residue levels.

Regulation of biocidal active ingredients and products proceeds via a similar route; however, peer review of the Member State assessments of the active ingredients are conducted by ECHA rather than EFSA. In 2017, ECHA and EFSA signed a memorandum of understanding to enhance cooperation between the agencies to facilitate coherence in scientific methods and opinions, and to share knowledge on matters of mutual interest. As a consequence, both agencies will evaluate the toxicological data package for a PPP.

#### 4.3.1 General requirements

Similar to the US and Canada, there are a large number of up-front data requirements required to register a plant protection active ingredient in the EU, including studies to assess potential hazards to humans and non-target organisms. The toxicology data requirements for support of an active ingredient or PPP are listed in Commission Regulation (EU) No 283/2013 and Commission Regulation (EU) No. 284/2013, respectively, and can be fulfilled using OECD test guideline studies or other guidelines (such as US EPA guidelines) that address the toxicological endpoint of concern. A number of data requirements, such as *in vivo* neurotoxicity studies or two-year rodent cancer bioassays in a second species, are only required when triggered or with scientific justification.

#### 4.3.2 Regulatory flexibility

Article 62(1) of Regulation (EC) No 1107/2009 requires that “testing on vertebrate animals for the purposes of this Regulation shall be undertaken only where no other methods are available.” Article 8(1)(d) and Article 33(3)(c) of the same Regulation requires applicants to justify, for each study using vertebrate animals, the steps taken to avoid testing on animals or duplication of studies. Similarly, for biocides, Article 62(1) of Regulation (EC) No 528/2012 states that “[i]n order to avoid animal testing, testing on vertebrates for the purposes of this Regulation shall be undertaken only as a last resort.”

The Commission Regulations, which list the data requirements for plant protection active ingredients and products, and their respective Communications (EU, 2013a; EU, 2013b) were published in 2013 and therefore only refer specifically to a limited number of NAMs (e.g., *in vitro* and *ex vivo* methods to assess skin irritation and eye irritation).

Although point 5.2 in the Annex of both Commission Regulation (EU) No 283/2013 and 284/2013 allow for the use of other NAMs, as they become available, to replace or reduce animal use, the outdated list of methods to fulfil data requirements in the Commission Communications may encourage animal use where NAMs should be used. For example, the methods listed to fulfil the requirements for skin sensitization do not include any of the available *in chemico* or *in vitro* methods and do not refer to the OECD Guideline on Defined Approaches to Skin Sensitization (EU, 2013a; EU, 2013b; OECD, 2021a). Therefore, the Commission Communications need to be updated urgently and regularly to avoid the use of animals.

As outlined above, the regulatory landscape of the EU is one of specific regional considerations and interpretation of legislation by individual Member States. For example, some Member State regulatory authorities responsible for PPPs, including those from the Czech Republic (SZU, n.d.), Sweden (KEMI, 2021), and Slovenia (Republika Slovenija, 2022), publicly align themselves with the legal requirement to justify the conduct of studies using vertebrate animals. Other Member States regulatory authorities, including those from the Netherlands (Ctgb, n.d.) and, pre-Brexit, the United Kingdom (HSE, n.d.), interpret the regulation more rigorously and state that applicants or dossiers will not be considered if they are found to have breached Article 62 (testing on vertebrate animals as a last resort).

#### 4.3.3 Implementation of NAMs

EFSA has been proactive in reducing animal testing and implementing reliable NAMs. For example, in 2009, EFSA published a scientific opinion covering the key data requirements for evaluation of pesticide toxicity that were amendable to NAMs (EFSA, 2009). In 2012, EFSA initiated a series of scientific conferences to create a regular opportunity to engage with partners and stakeholders. Following its latest conference in 2018 and the break-out session “Advancing risk assessment science—human health,” Lanzoni et al. have emphasized that the human health risk assessment based on animal testing is challenged scientifically and ethically (Lanzoni et al., 2019). They further mention the need for a paradigm shift in hazard and risk assessment and more flexible regulations.

EFSA has developed a chemical hazards database “OpenFoodTox 2.0” and funded collaborative research to develop generic toxicokinetic and toxicodynamic human and animal models to predict the toxicity of chemicals (Dorne et al., 2018; Benfenati et al., 2020). Further, in 2019, EFSA published their opinion on the use of *in vitro* comparative metabolism (IVCM) studies for use in pesticide risk assessment (EFSA, 2019). Currently, the IVCM study is a data requirement for new and renewal data packages being submitted in the EU. This study is intended to identify unique human metabolites as it compares to OECD TG 417, the toxicokinetic study currently performed in rats. Most recently, EFSA published their 2027 Strategy in which they state their goal, to develop and integrate NAMs for regulatory risk assessment (EFSA, 2021). To help achieve this, EFSA launched a contract to develop a roadmap for action on NAMs to reduce animal testing (Escher et al., 2022). The roadmap aims to define EFSA’s priorities for the incorporation of NAMs as well as to inform a multi-annual strategy for increasing the use of NAMs in human health risk assessment with a goal of minimizing animal testing (EFSA, 2021). In addition, EFSA is in the process of developing guidance on the use of read-across and has launched several projects to evaluate NAMs in the context of IATA frameworks.

##### 4.3.3.1 Examples of NAM application

EFSA has funded the development of *in vitro* assays that, together with the assays from ORD, form the current DNT NAM testing battery (see Section 4.1.3.1). In partnership with the OECD, EFSA held a workshop in 2017 on integrated approaches for testing and assessment of DNT (EFSA and OECD, 2017), commissioned an external scientific report on the data interpretation from *in vitro* DNT assays (Crofton and Mundy, 2021), and recently held a European stakeholder’s workshop on NAMs for DNT (EFSA, 2022). In 2021, the EFSA Panel on Plant Protection Products and their Residues (PPR) developed AOP-informed IATA case studies on DNT risk assessment (EFSA PPR Panel et al., 2021). The development of a new OECD Guidance Document on DNT *in vitro* assays is being co-led by EFSA, the US, and Denmark (OECD, 2021b).

In 2017, EFSA updated its guidance on dermal absorption that was initially published in 2012. The guidance presents elements for a tiered approach including “*in vitro* studies with human skin (regarded to provide the best estimate)” (EFSA et al., 2017), thereby reducing the use of animals while also increasing the relevance of the data for human risk assessment.

Furthermore*, in silico* modeling software, data mining, and read-across can be used for a variety of applications in support of pesticide registrations within the EU. Specifically, OECD-Toolbox, Derek Nexus, and OASIS TIMES are often used for the evaluation of toxicological significance of metabolites and impurities, and in support of active ingredient conclusions, especially related to genotoxicity (Benigni et al., 2019).

## 5 Conclusion

Due to widespread interest in the use of testing approaches that are reliable and relevant to human biology, NAMs for hazard and risk assessment are being rapidly developed. It is important to understand the existing regulatory frameworks, and their flexibility or limitations for the implementation of fit for purpose NAMs. This article provides an overview of the regulatory frameworks for the use of NAMs in the assessment of industrial chemicals and pesticides, in the US, Canada, and EU. However, similar collaborative efforts and opportunities to use NAMs in regulatory submissions exist in other sectors and countries. In general, replacing animal use is an important goal for regulatory agencies and, as such, regulators continue to explore the potential of NAMs to efficiently provide more reliable and relevant information about whether and how a chemical may cause toxicity in humans. The regulations reviewed in this paper highlight the many existing opportunities for the use of NAMs, while also showing potential to introduce further flexibility in testing requirements to allow the maximum use of fit for purpose NAMs.

For example, it is important to provide continuing educational opportunities for regulators and stakeholders on the conditions under which application of a certain NAM is appropriate and on how data from that NAM is interpreted. Conferences and webinars, as mentioned in Section 2, are examples of such opportunities. There are also ongoing discussions on how to streamline and accelerate validation processes and gain scientific confidence in the use of robust NAMs, including an ongoing effort within ICCVAM to publish a guidance on this topic. Updating these processes is foundational to timely uptake of fit-for-purpose, reliable, and relevant NAMs (van der Zalm et al., 2022). Also key to the advancement of NAMs is the opportunity to discuss proposed NAM testing strategies with the agency. This allows for the wise use of resources and ensures that data needs of the regulatory agencies are being addressed by the proposed approach. Each regulatory agency has varying ability and instructions on meeting with stakeholders to discuss proposed testing strategies, with some agencies (notably the EPA and HC’s NSP) strongly encouraging these meetings, resulting in examples of successful submissions. Additional measures to instate incentives, such as expedited review, would further facilitate innovation and the use of more modern, reliable NAMs.

In addition, national and international communication and collaboration within and across sectors and geographies is of the utmost importance to minimize duplicative efforts and efficiently advance the best science. Ultimately, regulatory frameworks that allow for the timely uptake of scientifically sound toxicology testing approaches will facilitate the global acceptance of NAMs and allow the best protection of human health.

## Footnotes

1 The distinction between an order and a rule is that the former may be issued without following the procedural requirements of notice and comment rulemaking under the Administrative Procedure Act (5 U.S.C. §§500 *et seq*.), whereas the latter must comply with these requirements.

## Glossary

AOP: Adverse Outcome Pathways
APCRA: Accelerating the Pace of Chemical Risk Assessment
BraCVAM: Brazilian Centre for the Validation of Alternative Methods
CaCVAM: Canadian Centre for the Validation of Alternative Methods
CATSAC: Chemistry and Acute Toxicology Science Advisory Council
CCA: Council of Canadian Academies
CCAAM: Canadian Centre for Alternatives to Animal Methods
CEPA: Canadian Environmental Protection Act
CFR: Code of Federal Regulations
CLP: Classification, labelling and packaging of substances and mixtures
CMP: Chemicals Management Plan
CPSC: US Consumer Products Safety Commission
CR: Conditionally required
CSS: EU Chemicals Strategy for Sustainability
DACO: Data-code
DAF: Dermal absorption factor
DNT: Developmental neurotoxicity
DSL: Domestic Substances List
EC: European Commission
ECHA: European Chemicals Agency
EFSA: European Food Safety Authority
EPA: US Environmental Protection Agency
ESRAP: Existing Substances Risk Assessment Program
EU: European Union
EURL ECVAM: EU Reference Laboratory for alternatives to animal testing
FD&C: Federal Food, Drug, and Cosmetic Act
FHSA: Federal Hazardous Substances Act
FIFRA: Federal Insecticide, Fungicide, and Rodenticide Act
FQPA: Food Quality Protection Act
GHS: Globally Harmonised System of Classification and Labelling of Chemicals
GLP: Good Laboratory Practice
HASPOC: Hazard and Science Policy Council
HC: Health Canada
HCI: High-content imaging
HECSB: Healthy Environments and Consumer Safety Branch
IATA: Integrated Approaches to Testing and Assessment
ICATM: International Cooperation on Alternative Test Methods
ICCVAM: US Interagency Coordinating Committee for the Validation of Alternative Methods
ITC: Interagency Testing Committee

IVCM: *In vitro* comparative metabolism

JaCVAM: Japanese Centre for the Validation of Alternative Methods
KoCVAM: Korean Centre for the Validation of Alternative Methods

MAD: Mutual Acceptance of Data
MEA NFA: Microelectrode array network formation array
NAFTA TWG: North American Free Trade Agreement Technical Working Group
NAM: New Approach Methodologies
NICEATM: US National Toxicology Program (NTP) Interagency Center for the Evaluation of Alternative Toxicological Methods
NRC: National Resource Council
NSN: New Substances Notification
NSNR: New Substances Notification Regulations
NSP: New Substances Program
NTP: US National Toxicology Program
OCSPP: Office of Chemical Safety and Pollution Prevention
OECD: Organisation for Economic Co-operation and Development
OPP: Office of Pesticide Programs
OPPT: Office of Pollution Prevention and Toxics
ORD: Office of Research and Development
PCPA: Pest Control Products Act
PMN: Pre-Manufacture Notice
PMRA: Pest Management Regulatory Agency
PPP: Plant Protection Products
PPR: EFSA Panel on Plant Protection Products and their Residues
QSAR: Quantitative Structure-Activity Relationship
RCC: Canada-US Regulatory Co-operation Council
REACH: Registration, Evaluation, Authorisation and Restriction of Chemicals
ReCAAP: Rethink Chronic toxicity and carcinogenicity Assessment for Agrochemicals
SAP: Scientific Advisory Panel
SC: Statutes of Canada
SciAD: Science Approach Documents
SIEFs: Substance Information Exchange Fora
SNUN: Significant New Use Notice
SNUR: Significant New Use Rule
SOR: Statutory Orders and Regulations
TG: Test Guidelines
TSAR: Tracking System for Alternative Methods
TSCA: Toxic Substances Control Act
TTC: Threshold of Toxicological Concern
UNECE: United Nations Economic Commission for Europe
US: United States
USC: US Code
USMCA: US-Mexico-Canada Agreement
WoE: Weight of (scientific) Evidence
